# In vivo applications and toxicities of AAV-based gene therapies in rare diseases

**DOI:** 10.1186/s13023-025-03893-z

**Published:** 2025-07-17

**Authors:** Qian Zhao, Huifang Peng, Yujin Ma, Huijun Yuan, Hongwei Jiang

**Affiliations:** 1 Luoyang Key Laboratory of Clinical Multiomics and Translational Medicine, Henan Key Laboratory of Rare Diseases, Endocrinology and Metabolism Center, The First Affiliated Hospital, College of Clinical Medicine, Henan University of Science and Technology, Luoyang, 471003 China; 2 Institute of Rare Diseases, West China Hospital of Sichuan University, Chengdu, 610000 China

**Keywords:** Adeno-associated virus, Clinical trials, Efficacy, Adverse effects/Toxicities, Gene therapies

## Abstract

Adeno-associated virus (AAV), renowned for its exceptionally low pathogenicity and significant efficacy in clinical gene therapy, has emerged as a leading delivery vector in the field of gene therapy. AAV can achieve stable gene expression in various tissues, which has made it a promising treatment for genetic disorders. To date, eight AAV-based gene therapies have been approved by the U.S. Food and Drug Administration (FDA) and European Medicines Agency (EMA). This review summarizes clinical trials of AAV gene therapies for rare diseases, including ophthalmic diseases, nervous system disorders, hematological diseases, neuromuscular diseases, lysosomal storage diseases. We also explore potential side effects and toxicities associated with AAV therapies. Our objective is to provide valuable insights for researchers and clinicians working on AAV-based therapies, helping improve the safety and effectiveness of these treatments.

## Introduction

Gene therapies refer to strategies for treating diseases through the introduction of exogenous therapeutic genes into target cells or tissues via nonviral or viral vectors [1]. Viral surface proteins can effectively recognize cell receptors and transport genetic material into host cells, where the viral genome is maintained episomally and the transgene is expressed over an extended period. In some cases, the expression of the transgene can be regulated by the incorporation of specific regulatory elements, allowing for targeted expression in particular cell types or under specific conditions [2]. Consequently, the use of viral vectors has significantly advanced the conceptual exploration and clinical application of gene therapy. In recent years, AAV-based gene therapies have emerged as a popular therapeutic option because of the structural simplicity, safety, and versatility of molecular manipulation of AAV, and several of these therapies have been approved by the FDA [3]. Given the high unmet clinical need in rare diseases, understanding the clinical applications and toxicities of AAV-based gene therapies is essential.

### The manufacture of AAV vectors

**Fig. 1:**
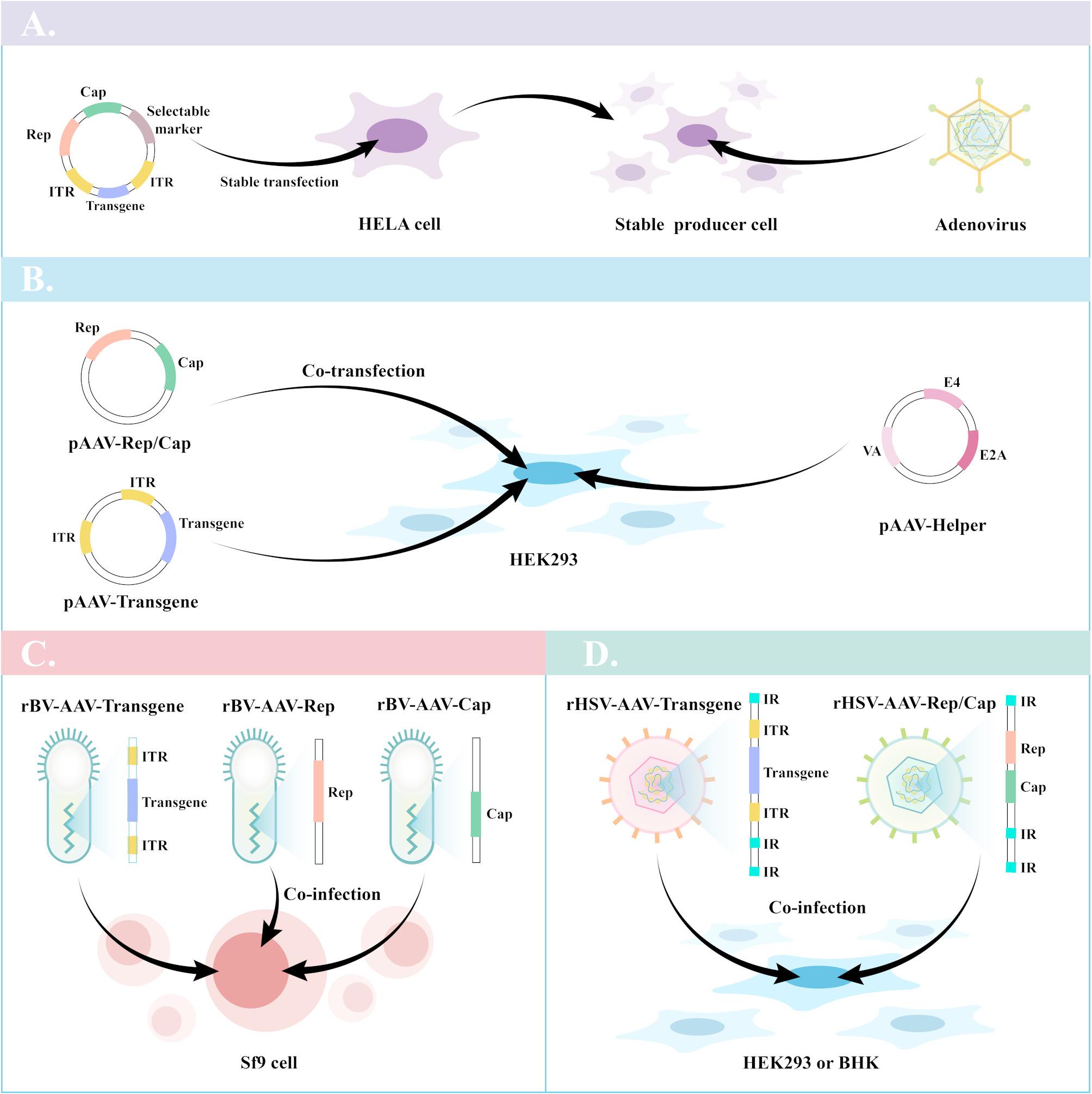
Current approaches to manufacture rAAV. **(A)** Stable transfection. **(B)** Co-transfection. **(C)** Co-infection (Sf9 cells co-infected with rBVs vectors). **(D)** Co-infection (HEK293 cells or BHK cells co-infected with two rHSV vectors)

AAV is a small, single-stranded DNA virus with a genome of about 4.7 kb, containing inverted terminal repeats (ITRs). The genomic structure of AAV consists mostly of two open reading frames (ORFs), rep and cap. The cap gene encodes proteins VP1, VP2, and VP3, which form the viral capsid and help with cell attachment and internalization. The rep gene produces proteins needed for replication. AAV’s icosahedral shell consists of 60 subunits and determines its serotype-specific function, with at least 13 recognized serotypes, each of which exhibits different tissue tropisms (Table 1) [4]. The assembly-activating protein (AAP), encoded by the aap gene in an alternate reading frame that overlaps across the cap gene, helps stabilize and assemble the capsid [5]. The production of recombinant AAV (rAAV) vectors involves two main strategies: stable and transient expression. In stable expression, HeLa cells are transfected with a plasmid that includes marker genes, AAV rep and cap genes, and recombinant vector genomes to create producer cell lines (Fig. 1A) [6]. These cell lines are then infected with adenoviruses to generate infectious rAAV. For transient expression, HEK293 cells are transfected with three plasmids: a transgene plasmid (containing ITRs and target genes), a rep/cap plasmid, and a helper plasmid (Fig. 1B) [7]. Alternatively, rAAV can be expressed in insect cells like Spodoptera frugiperda (Sf9) by co-infecting them with recombinant baculovirus vectors containing the AAV components (Fig. 1C) [8]. Another method involves using recombinant herpes simplex virus (rHSV) vectors to infect HEK293 or BHK cells and produce rAAV (Fig. 1D) [9].

**Table 1:** The tissue tropisms of different AAV serotypes

AAV Serotype	Tissue-Specific Tropisms
AAV1	Muscle, heart, skeletal muscle (including cardiac muscle), nerve tissue
AAV2	Central nervous system, muscle, liver, brain tissue, eye
AAV3	Muscles, liver, lung, eye
AAV4	Central nervous system, muscle, eye, brain
AAV5	Lung, eye, central nerve, joint synovium, pancreas
AAV6	Lung, heart
AAV7	Muscle, liver
AAV8	Liver, eye, central nerve, muscle
AAV9	Heart, muscle, lung(alveolar), liver, central nervous system
AAV10	Lung, heart, muscle, central nervous system, liver
AAV11	Unknown
AAV12	Nasal
AAV13	central nervous system

### Administration route of AAV-based gene therapy

Unlike conventional drug therapy, the route of administration of gene therapy can significantly affect therapeutic efficacy and the potential for adverse events. The same vector can be tested with different paths and produce different results. The selection of the drug delivery route should be based on the characteristics of the disease and the transduction efficiency of the AAV capsid.

**Fig. 2:**
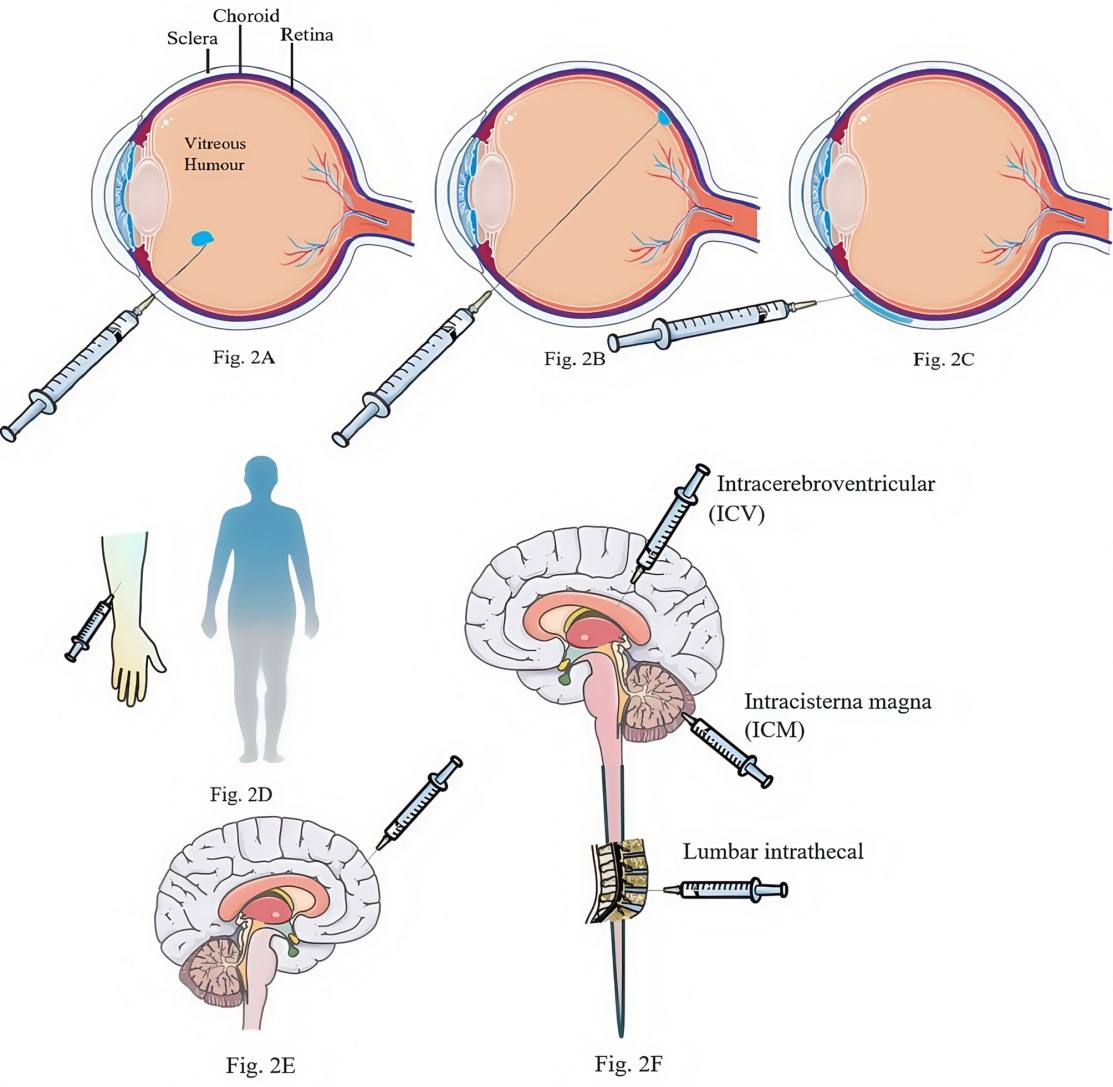
Routes of administration for gene therapy. **(A)** Intravitreal Injection. **(B)** Subretinal Injection. **(C)** Suprachoroidal Injection. **(D)** Intravenous (IV) administration. **(E)** Intraparenchymal (IPa) administration. **(F)** Intra-cerebrospinal fluid (intra-CSF) administration

Gene therapy vectors for ophthalmic diseases can be introduced into the retina in three ways (Fig. 2). Intravitreal injection (Fig. 2A) is a less invasive method in which the vector is injected into the vitreous cavity through the pars plana [10]. While this route allows better access to cells inside the retina, it also results in less efficient transduction of cells outside the human retina owing to the barrier formed by the internal limiting membrane (ILM) and the neuroretina [11]. Subretinal injection (Fig. 2B) allows better penetration of the retinal pigmented epithelium (RPE) and photoreceptors, which are commonly affected by inherited retinal disease (IRD) [12]. However, subretinal injection requires a greater level of surgical skill. Suprachoroidal injection (Fig. 2C) is a minimally invasive drug delivery route in which, in contrast to traditional vitreous or subretinal injections, the entire wall of the eye does not need to be penetrated—only the outermost scleral layer—therefore, the seal and integrity of the eye is maintained. This approach reduces the risk of intraocular bleeding and eye tissue damage while also reducing the risks associated with off-target effects of gene products, increasing the safety and bioavailability of drugs [13].

The systemic route of administration, such as intravenous (IV) administration, is a common method of drug delivery and is suitable for situations where efficacy needs to be achieved quickly or for diffuse treatment of central nervous system (CNS) and peripheral tissues. IV administration allows the drug to enter the bloodstream directly and rapidly distribute throughout the body, including CNS regions such as the brain and spinal cord, as well as peripheral tissues (Fig. 2D). However, recent clinical studies have shown that an AAV serotype that can efficiently cross the blood‒brain barrier is required for IV administration, as are higher doses, which may lead to associated toxicities [14].

Intraparenchymal (IPa) administration is a local route of administration in which drugs are delivered directly into brain tissue (Fig. 2E). This approach is suitable for diseases associated with dysfunction in specific brain regions, especially cell-autonomous diseases, which may not require systemic treatment or greater side effects may occur from systemic treatment, such as aromatic L-amino acid decarboxylase deficiency (AADCD) and Parkinson’s disease (PD). The advantage of IPa administration is that biological distribution to surrounding organs can be minimized, which may limit the overall immunogenicity of the vector and significantly reduce the vector dose required [15]. The intracerebroventricular (ICV), intracisterna magna (ICM), and lumbar intrathecal routes of administration (Fig. 2F) can be used to achieve a wide range of CNS transduction. Direct injection into the cerebrospinal fluid (CSF) can bypass the blood‒brain barrier, thereby allowing higher transgene copy numbers to be delivered to the brain and reducing off-target gene product transfer to peripheral organs such as the liver. Another benefit of intravascular administration is that some degree of preexisting circulating anti-AAV antibodies can be tolerated with this method [16].

### Clinical applications of AAV-based gene therapy in rare diseases

As of early 2024, eight AAV-based gene therapy drugs have been approved [17–24] (Table 2). Notably, Glybera (AAV1), the first AAV therapy approved by both the FDA and EMA in 2012 for lipoprotein lipase deficiency (LPLD), was discontinued in 2017 owing to prohibitive treatment costs and a limited eligible patient population [17]. Here, we examine the application of AAV-based gene therapies in ongoing clinical studies for neurological, ophthalmic, metabolic, hematological, neuromuscular, and cardiovascular disorders. The aim of this study is to provide researchers and clinicians with useful guidance for administering AAV-based gene therapies.

**Table 2:** Approved AAV-based gene therapy drugs

Drug name	Time and organization of approval	Administration	Payload (Gene)	Mechanism of Action	Delivery vector	Indication
Glybera	2012(EMA)	Intramuscular Injection	lipoprotein lipase (LPL) gene	Glybera delivers the functional LPL gene to muscle cells via an AAV1 vector, enabling LPL enzyme production to break down blood chylomicrons and reduce hypertriglyceridemia in patients with LPLD.	AAV1	LPLD
Luxturna	2017(FDA) 2018(EMA)	Subretinal injection	RPE65 gene	Luxturna delivers the functional RPE65 gene to retinal cells via an AAV2 vector, compensating for the enzyme deficiency caused by mutations, restoring the visual cycle, and thereby improving or stabilizing patients’ vision.	AAV2	IRD
Zolgensma	2019(FDA) 2020(EMA)	Intravenous Injection	SMN1 gene	Zolgensma utilizes an AAV9 vector to deliver a functional SMN1 gene to motor neurons, restoring SMN protein expression and halting neurodegenerative progression in SMA, thereby improving motor function.	scAAV9	SMA
Upstaza	2022(EMA)	Intraparenchymal Injection	DDC gene	Upstaza delivers the functional DDC gene to the basal ganglia region in the brain via an AAV2 vector, restoring AADC enzyme activity, promoting dopamine and serotonin synthesis, and improving neurological function in patients with AADCD.	AAV2	AADCD
Hemgenix	2022(FDA) 2023(EMA)	Intravenous Injection	FIX-Padua variant	Hemgenix uses an AAV5 vector to deliver an optimized FIX gene (Padua variant) to liver cells, enabling sustained production of high-activity factor IX to restore blood clotting and reduce bleeding risk in Hem B patients, minimizing dependence on exogenous factor replacement therapy.	AAV5	Hem B
Roctavian	2022(EMA) 2023(FDA)	Intravenous Injection	B-domain-deleted FVIII	Roctavian utilizes an AAV5 vector to deliver the functional FVIII gene to liver cells, enabling sustained production of factor VIII to restore blood clotting and reduce bleeding episodes in Hem A patients, decreasing reliance on exogenous FVIII infusions.	AAV5	Hem A
Elevidys	2023(FDA)	Intravenous Injection	Micro-dystrophin gene	Elevidys uses an AAVrh74 vector to deliver a functional micro-dystrophin gene to muscle cells, restoring partial dystrophin protein expression to stabilize muscle membranes and slow disease progression in DMD patients.	AAVrh74	DMD
Beqvez/ Durveqtix	2024(FDA/EMA)	Intravenous Injection	Highly active FIX variant	Beqvez/Durveqtix delivers a high-activity variant of the FIX gene via an AAVRh74var vector, allowing patients to produce their own FIX protein, thereby reducing or eliminating the need for regular intravenous FIX infusions in HemB patients.	AAVRh74var	Hem B

### Ophthalmic diseases

#### Inherited retinal disease (IRD)

Leber’s congenital amaurosis (LCA) is a serious, early genetic retinal disease that is characterized by severe visual impairment or blindness, nystagmus, and a dulled or lost pupil reflex to light. LCA has a high degree of genetic heterogeneity, and the typing is based on different genetic mutations, and LCA2 is associated with mutations in the RPE65 gene [25]. Luxturna is an AAV2-based gene therapy that delivers the RPE65 gene to retinal cells. A Phase III trial involving 29 LCA2 patients demonstrated that Luxturna significantly improved functional vision after one year compared to no treatment [26]. The intervention group showed over a 100-fold improvement in vision by day 30, with the benefits sustained for more than a year. Follow-up studies indicated that these improvements persisted for 3 to 4 years [27, 28]. This study led to Luxturna (voretigene neparvovec) being approved by the FDA for the treatment of patients with confirmed biallelic RPE65 mutation-associated IRD [18].

Retinitis pigmentosa (RP) is an IRD characterized by the gradual degeneration of photoreceptor cells in the retina, resulting in decreased vision and a reduced field of vision [29]. RP is highly heterogeneous, and RPE65 gene mutations are among the known causes of RP. Luxturna has been approved by the FDA for the treatment of RPE65 mutation-associated IRD. Given the diversity of disease-causing mutations in RP, multiple gene therapy strategies have been implemented. In a phase I trial conducted in 2016, the results revealed that subretinal administration of AAV2-hMERTK was safe in 6 patients with MERTK-related RP, and vision gain was maintained for only 1 patient at the 2-year follow-up [30]. The first phase I/II dose-escalation clinical trial was for the treatment of X-linked RP caused by RPGTPase regulatory factor mutations, with a total of 18 patients receiving subretinal delivery of cotoretigene toliparvovec (AAV8-hRPGR). In a subset of patients, neither dose-limiting toxicity nor a restored visual field was observed [31]. The results of this trial confirmed the effectiveness of cotoretigene toliparvovec at 1 year after injection compared with no treatment [32]. The 4 patients who received the highest dose of the drug had early and consistent benefits in terms of visual function during treatment when compared with 1 patient who received no treatment.

### Neurological diseases

#### AADCD

AADCD, an autosomal recessive disorder, is caused by mutations in the dopamine decarboxylase (DDC) gene encoding AADC, resulting in significant defects in dopamine and serotonin synthesis [33]. Children suffering from severe disease are unable to achieve normal developmental goals and unable to complete movements such as raising their head, sitting or standing. These patients usually have a life expectancy of no more than 5 to 6 years.

The efficacy and safety of AAV2-based gene therapy for AADCD have been validated in multiple clinical trials. In 2012, four patients aged 4–6 years received an intraputaminal infusion of 1.6 × 10^11^ vg AAV2-hAADC, leading to improvements in cognitive and motor function, increased dopamine and serotonin levels, and enhanced AADC uptake in the putamen, with no immune response observed in 50% of patients [34]. In subsequent Phase I/II trials, 10 children aged 2–6 years demonstrated good tolerability and motor development improvements, with effects lasting over five years [35, 36]. A 2019 trial (six patients aged 4–19 years, 2 × 10^11^ vg dose) further confirmed a significant increase in dopamine levels within 12 months [37]. Eladocagene exuparvovec (Upstaza), an AAV2-based therapy, restores AADC enzyme activity in the putamen by delivering a functional DDC gene. Its safety and efficacy were validated across three consecutive trials (compassionate use, Phase I/II, and Phase IIb) involving 26 patients. Rapid improvements in motor and cognitive function were observed within 12 months and sustained for over five years, with no treatment-related brain injuries reported [36]. On the basis of these results, Upstaza was officially approved by the EMA in 2022 for the treatment of AADCD in patients older than 18 months [20].

### Spinal muscular atrophy (SMA)

SMA is an autosomal recessive destructive neurodegenerative disease in which 95% of patients have deletion or mutation of the spinal cord motor neuron 1 (SMN1) gene, resulting in insufficient secretion of the functional SMN protein [38] and triggering progressive loss of motor neurons. The SMN2 gene is homologous to SMN1, produces the smallest quantity of SMN protein and has numerous copies [39]. There is a correlation between the number of copies of SMN2 and the onset and severity of SMA [40]. The most common variant is SMA1; affected individuals have two copies of SMN2, and this variant can lead to death before age 2 or dependence on mechanical ventilation to stay alive.

In 2017, the first clinical trial included 15 patients with SMA1 who were given intravenous infusions of 6.7 × 10^13^ vg/kg or 2.0 × 10^14^ vg/kg AAV9-hSMN. The results showed that patients had fewer requirements for breath-ventilator treatment and improved motor scores [41]. In two phase III trials of Zolgensma (AAV9-hSMN), patients aged less than 6 months who had symptomatic SMA1 were treated with a dose of 1.1 × 10^14^ vg/kg (STR1VE-US, *n* = 22; STR1VE-EU, *n* = 33) [42, 43]. Both studies showed that intravenous injection of Zolgensma for SMN gene replacement increased survival and promoted the development of movement in SMA1 patients. At 14 months of age, 91% (STR1VE-US) and 97% (STR1VE-EU) of patients who were not on permanent mechanical ventilation were alive. Additionally, both trials revealed consistent and rapid improvements in motor function. The SPR1N study assessed the efficacy and safety of presymptomatic SMN gene therapy (Zolgensma, 1.1 × 10^14^ vg/kg) in children genetically diagnosed with spinal muscular atrophy (SMA), hypothesizing earlier intervention improves outcomes. It involved 14 children with 2 SMN2 copies [44] and 15 with 3 SMN2 copies [45]. At 18 months, all 14 patients with 2 copies achieved independent sitting (≥ 30 s), 13 maintained stable weight (≥ 3rd percentile per WHO standards), while 15 children with 3 copies stood independently by 24 months, and 14 walked independently. No patients required mechanical ventilation by 14 months, all survived, and the weight of ten patients (67%) was maintained without feeding support. Neither cohort needed nutritional/respiratory support, and no treatment-related serious adverse events occurred, demonstrating the advantages of early presymptomatic therapy. In 2019, the FDA officially approved Zolgensma for the treatment of SMA1 in children under 2 years of age [19].

### Canavan disease (CD)

CD is a leukodystrophy caused by a pathogenic mutation of aspartate acylase (ASPA). ASPA deacetylates and reduces n-acetylaspartic acid (NAA). Excess NAA can lead to parenchymal edema, vacuole formation, and abnormal myelination of white matter [46]. In a phase I study, AAV2-hASPA was administered at a dose of 1 × 10^12^ to 10 patients with CD; no neutralizing antibodies against rAAV2 were detected in CSF, and minimal to mild systemic immune responses were detected, underscoring the relative safety of AAV2-hASPA [47]. On the basis of the above study findings, 28 patients with CD were enrolled in a phase I/II clinical trial, and the long-term safety of AAV2-hASPA treatment was evaluated [48]. The results showed that NAA levels in the CNS were decreased, the progression of brain atrophy slowed, the number of seizures decreased, and the disease phenotype was generally stable.

### Tay‒Sachs disease (TSD)

TSD is an inherited neurological disorder characterized by autosomal recessive mutations in the gene encoding the α-subunit of hexosaminidase A (HexA) [49]. Currently, there is no cure, but research in gene therapy is progressing. Two patients with TSD received AAV-based gene therapy in a clinical trial [50]. Patient TSD-001 received a combination of equal doses of AAVrh8-HexA and AAVrh8-HexB at 30 months, with 75% of the total dose (1 × 10^14^ vg) in the cerebellar bulbar cisterna and 25% at the thoracolumbar junction. Patient TSD-002 was treated at 7 months with a combination of AAVrh8-HexA and AAVrh8-HexB administered in the bilateral thalamus (1.5 × 10^12^ vg) and via intravenous drip (3.9 × 10^13^ vg). The injection procedure was well tolerated, and no vector-related adverse events occurred. HexA activity in the CSF increased from baseline and remained stable in both patients. Three months after injection, patient TSD-002 showed stable disease with persistent myelination and a temporary deviation from the natural history of TSD in infants, but disease progression was evident at six months after treatment. With the same anticonvulsant medication as before treatment, patient TSD-001 remained seizure free at 5 years of age. Patient TSD-002 developed anticonvulsant reactive seizures at 2 years of age. This study provides early safety and proof-of-concept data for AAV-based gene therapy in patients with TSD.

### Blood diseases (nontumor)

Hemophilia is an X-linked recessive genetic disorder. HemA accounts for approximately 85% of hemophilia cases and is caused by factor VIII (FVIII) deficiency. HemB is caused by factor IX (FIX) deficiency. A lack of FVIII or FIX prevents the activation of factor X (part of the common pathway), resulting in reduced activation of thrombin and fibrin, which ultimately affects clot formation. HemA or B are classified by FVIII or FIX activity levels: (50–150 IU/dL, normal; <1 IU/dL, severe; 1–5 IU/dL, moderate; and 6–40 IU/dL, mild) [51]. The treatment goal for people with hemophilia is to maintain sufficient levels of factor activity to prevent bleeding that can lead to devastating joint disease or major organ disease, which can lead to death.

### Hemophilia A (HemA)

Valoctocogene roxaparvovec (Roctavian) expresses the B-domain deficient (BDD) FVIII-SQ variant (AAV5-hFVIII-SQ) via the AAV5 vector. The phase I/II dose-escalation study (NCT02576795) enrolled 9 adult males with severe Hemophilia A (HemA), administering low (6 × 10^12^ vg/kg, *n* = 1), medium (2 × 10^13^ vg/kg, *n* = 1), and high doses (6 × 10^13^ vg/kg, *n* = 7). While low/medium doses maintained FVIII activity ≤ 3 IU/dL, all high-dose patients achieved > 5 IU/dL, with 6/7 reaching normal levels (> 50 IU/dL) sustained for 1 year [52]. The study identified a 1.6-fold higher FVIII measurement via one-step analysis (OSAs) versus chromogenic substrate assays (CSAs), highlighting methodological discrepancies critical for cross-trial comparisons [53]. In the phase III trial (NCT03370913), 134 adult HemA patients received 6 × 10^13^ vg/kg Roctavian [54]demonstrating median chromogenic FVIII activity of 22.9 IU/dL, sustained bleeding reduction, and eliminated prophylactic FVIII use, with therapeutic durability and safety maintained for ≥ 2 years post-treatment [55]. Roctavian was approved by the EMA in 2022 and by the FDA in 2023 for the treatment of eligible patients with severe HemA [21].

### Hemophilia B (HemB)

Etranacogene dezaparvovec (Hemgenix) delivers the FIX gene to liver cells via the AAV5 vector, causing the cells to produce the missing FIX. In the phase IIb clinical trial of Hemgenix (AAV5-hFix-Padua), FIX activity in three patients with AAV5 antibodies increased to an average of 40.8% in the first year after administration and remained at 36.9% in the third year [56]. In the phase III study, we analyzed participants who were seronegative (*n* = 31) or seropositive (*n* = 23) for AAV5 and found that they all presented FIX activity levels similar to those reported in the IIb study. This finding shows that even when patients’ levels of anti-AAV5 neutralizing antibodies are below the threshold, these patients are still able to respond positively to this gene therapy [57]. At three years after treatment, 51 patients (94%) had not received FIX prophylaxis. The average endogenous FIX activity of participants remained stable at year 1; there was a slight decrease in the second year, and in the third year after treatment, the level rose again [58].

Fidanacogene elaparvovec (Beqvez/Durveqtix) expresses a highly active variant of the FIX gene via the AAVRh74var vector, enabling patients to produce autologous FIX proteins through treatment and reducing or eliminating the need for regular intravenous infusions of FIX in patients. The phase I/IIa trial evaluating fidanacogene elaparvovec (5 × 10^11^ vg/kg) in 15 adults with moderate-to-severe hemophilia B (FIX ≤ 2%) demonstrated sustained efficacy and safety [59]: all participants completed the 52-week study, with 14 transitioning to the 5-year long-term follow-up (LTFU; 13 remaining as of December 2020). While three patients required corticosteroid therapy within the first 6 months, no LTFU participants needed corticosteroids or FIX prophylaxis reinstatement. No treatment-related serious adverse events (SAEs) occurred in the initial study, though three unrelated SAEs emerged during LTFU. Safety assessments revealed no antibodies, thrombotic events, or clinically significant liver mass/alpha-fetoprotein (AFP) increases. Four patients underwent surgery (including two emergency procedures) without bleeding complications or supplemental FIX, confirming durable therapeutic efficacy. On the basis of the stable expression of FIX in the phase I/IIa study, 45 men with AAV-seronegative HemB [60] were enrolled in a phase III study, which revealed a 71% reduction in the annualized bleeding rate. At 24 months, FIX activity averaged 25%, with 13.3% requiring prophylaxis due to immune response recovery and up to 62.2% needing corticosteroids initially. However, by the end of the first year, no patients required corticosteroid treatment.

On the basis of these study findings, Hemgenix [22] was approved for marketing by the FDA (2022) and EMA (2023). Beqvez/Durveqtix was approved by the EMA and FDA in 2024 for the treatment of eligible patients with HemB [24].

### Myopathy

#### Duchenne muscular dystrophy (DMD)

DMD is an X-linked degenerative neuromuscular disease caused by mutations in the DMD gene, resulting in a lack of functional muscular dystrophy protein [61]. SRP-9001 (AAVrh74-hMHCK7) is driven by a synthetic MHCK7 promoter, consisting of a creatine kinase (MCK) promoter fused with MCK and an α-myosin heavy chain complex (α-MHC) enhancer, promoting high expression levels in skeletal and cardiac muscle [62]. A phase I/IIa trial (NCT03375164) evaluated the safety and tolerability of SRP-9001 (Elevidys delandistrogene moxeparvovec) in patients with DMD [63]including 4 patients who received a single dose of 2.0 × 10^14^ vg/kg intravenously. No SAEs occurred in any of the patients. At 12 weeks, immunohistochemistry of gastrocnemius biopsy samples from all patients revealed strong transgene expression, with microdystrophin expressed in an average of 81.2% of muscle fibers, increased North Star Ambulatory Assessment (NSAA) scores in all patients, and reduced creatine kinase levels (compared with those at baseline after treatment) maintained for 1 year. Three of the treated patients underwent quantitative MRI and spectral analysis [64]which revealed improvements in muscle fat fraction and transverse relaxation time (qT2, affected by inflammation and fat infiltration) values in patients treated with SRP-9001 compared with the natural history cohort. Clinical trials of SRP-9001 are ongoing (phase II randomized placebo-controlled, NCT03769116; phase I, NCT04626674; and phase III double-blind, randomized, placebo-controlled, NCT05096221) [65, 66]and preliminary data are available through press releases and conference reports. On the basis of the results of their entire clinical program, Elevidys was approved by the FDA in 2023, becoming the first gene therapy for the treatment of DMD patients aged 4–5 years [23].

### Lysosomal storage disease

#### Gaucher disease (GD)

GD is a multisystemically involved autosomal recessive lysosomal storage disease [67]. Owing to the mutation of the glucocerebrosidase (GBA) gene, GBA activity in the body is deficient or decreased, resulting in the presence of the substrate glucocerebroside (GL-1) in the liver, spleen, kidney, bone, lung, liver, liver and liver. GL-1 is even stored in the macrophages of the brain. GD can be divided into three subtypes according to whether the nervous system is involved and the rate of progression—nonneuropathic variant (GD1), acute neuropathic variant (GD2), chronic or subacute neuropathic variant (GD3)—as well as rare subtypes, such as the perinatal lethal type.

There are no FDA-approved gene therapies for GD. Several clinical studies of GD1 based on the AAV vector are underway worldwide (Table 3): (1) The clinical trial of the AAV9 gene therapy PR001 (LY3884961) in the treatment of adult GD1 peripheral manifestations (NCT05487599) is still in the phase I/II clinical enrollment phase [68]. (2) The AAV8 gene therapy FLT201 for adult GD1 patients is still in the phase I clinical recruitment stage (NCT05324943) [69]. (3) A Chinese first phase I clinical trial (NCT06162338) of LY-M001 injection [70]an AAV-based gene therapy drug targeting GD1, is being conducted to evaluate the safety and initial efficacy of LY-M001 in the treatment of adult GD1. There is little global research on gene therapy for GD2. PR001, a gene therapy for GD1, was used to treat GD2 in infants and is currently in a phase I/II clinical trial (NCT04411654) [71]. The other study is a clinical trial of AAV9 gene therapy for GD2 at Shanghai Xinhua Hospital (NCT06272149) [72], which is currently underway. Currently, no AAV-based gene therapy clinical trials are being conducted for GD3. Gene therapy for GD is still being developed and explored, and this therapy is expected to provide more and better specific treatment options for GD patients in the future to improve clinical outcomes and quality of life.

**Table 3:** AAV-based gene therapies under development

Drug Name/Candidate	Indication	Payload (Gene)	Mechanism of Action	Delivery Vector
AAV2-hASPA	CD	ASPA gene	Restores ASPA enzyme activity, enabling breakdown of NAAA and reducing neurotoxic metabolite accumulation	AAV2
AAVrh8-HexA	TSD	HexA gene	Uses the AAVrh8 serotype vector to deliver the HEXA gene to neurons or hepatocytes, restoring HexA enzyme activity and degrading accumulated GM2 gangliosides	AAVrh8
PR001 (LY3884961)	GD1/GD2	GBA1 gene	PR001 delivers the functional GBA1 gene via an AAV9 vector, restoring glucocerebrosidase activity and breaking down accumulated substrates	AAV9
FLT201	GD1	GBA1 gene	FLT201 delivers the GBA1 gene to liver cells via a liver-targeted AAV8 vector, enabling sustained secretion of functional GBA to degrade accumulated glucosylceramide	AAV8
LY-M001	GD1	GBA1 gene	Restores glucocerebrosidase activity and breaking down accumulated substrates	AAV9
VGN-R08b	GD2	GBA1 gene	Restores glucocerebrosidase activity and breaking down accumulated substrates	AAV9
ST-920	FD	GLA gene	ST-920 delivers the GLA gene to liver cells via a liver-targeted AAV vector, enabling sustained secretion of functional α-Gal A to degrade accumulated toxic substrates GL-3	AAV2/6

#### Fabry disease (FD)

FD is an X chromosome-linked hereditary lysosomal storage disease. Owing to GLA gene mutation, the activity of α-galactosidase A (α-Gal A) is reduced or completely absent, resulting in the accumulation of the metabolic substrate trihexyl-sphingolipol (GL-3) and its derivative deacetylated GL-3 (Lyso-GL-3) in multiple organs. Multiple organ lesions can even lead to life-threatening complications [73].

ST-920 is a recombinant AAV2/6 vector encoding human α-Gal cDNA. The phase I/II clinical trial (NCT04046224) is evaluating the tolerability and safety of ST-920 [74], which is administered as a single infusion (cohort 1: 0.5e13 vg/kg, cohort 2: 1.0e13 vg/kg, cohort 3: 3.0e13 vg/kg, cohort 4: 5.0e13 vg/kg), with data published as of September 17, 2021. The results from the first two dose cohorts indicate that all 4 patients achieved α-Gal A activity 2–15 times higher than normal. α-Gal A activity levels were maintained for up to 1 year in the first patient. In one patient whose Lyso-GL-3 was significantly elevated before treatment, the Lyso-GL-3 level was significantly reduced by approximately 40% within 10 weeks of treatment with ST-920, and the effect was maintained until week 32. All patients tolerated treatment well, and no treatment-related SAEs occurred [75, 76]. Another long-term follow-up study evaluating patients treated with ST-920 is underway (NCT05039866) [77]. Although more investigations are needed to ascertain its clinical efficacy and safety, gene therapy for FD has potential for both tissue-directed therapy and long-term cure.

## Adverse effects and toxicities of AAV-based gene therapies

### Hepatotoxicity

Hepatotoxicity, defined as elevated liver transaminase and jaundice, is a common adverse event with AAV-gene based therapies [78]. Different AAV serotypes have a proclivity for different organs, but the liver is the main organ where most AAV serotypes accumulate [79]. In a clinical trial of the AAV5-hFVIII-SQ for HemA [54]115 participants (85.8%) experienced elevated alanine aminotransferase (ALT) levels. Among these patients, 12 (8.9%) experienced grade 3 events (> 520 times the upper limit of the normal range), but all patients recovered after treatment with glucocorticoids. In a clinical study on HemB, 11 participants (20%) had mildly or moderately elevated ALT levels after receiving Hemgenix [57]. Mildly or moderately elevated ALT levels were reported in 3 of 14 children (21%) with SMA after Zolgensma administration in 2022, which resolved after treatment [44]. In another study of four patients with DMD treated with Elevidys, three patients had elevated liver enzyme levels, which resolved with corticosteroid treatment [63]. In a clinical trial of AAV8-based gene therapy for X-linked myopathy, liver toxicity resulted in the death of four patients [80]. While the exact mechanisms that cause these toxicities are under investigation, one hypothesis attributes this effect to preexisting antibodies targeting AAV, leading to activation of the classic pathway of innate immunity and/or the complement system. The inflammatory response to AAV is dominated by CD^8+^ T cells, highlighting the role of T-cell populations in hepatotoxicity [81].

### Dorsal root ganglion (DGR) toxicity

In several trials in nonhuman primates, intravaginal, intraventricular, macrocisternal, and, to a lesser extent, intravenously transferred high-dose AAV vectors have been observed to cause DGR toxicity that is not attenuated by immunosuppression and is characterized by axonal degeneration, neuronal damage, and B and T-cell infiltration [82].

In one clinical trial [83]an intrathecal infusion of AAV-miR-SOD1 was administered to two patients suffering from familial amyotrophic lateral sclerosis (ALS) owing to mutations in the gene encoding superoxide dismutase 1 (SOD1). One patient treated with prednisolone on the day of gene therapy presented an increase in the number of circulating AAV capsule-specific T cells approximately 4 weeks after treatment, with neurological symptoms. MRI scan results were consistent with DRG toxicity. The second patient received a more aggressive immunosuppressive regimen that reduced the production of neutralizing antibodies and antiviral antibodies and the T-cell response to the viral capsid; no neurological symptoms were observed, and no significant abnormalities were seen on MRI. As more patients are treated with rAAV-based gene therapies, elucidating the mechanisms, diagnosis, prevention, and treatment of neurotoxicity is important to increase safety.

### Thrombotic microangiopathy (TMA)

TMA is thought to be caused by endothelial injury and associated platelet aggregation, leading to microthrombus formation, thrombocytopenia, end-organ damage through ischemia, and death. TMA can be acquired or can be inherited [84].

Of the 1,400 people treated with Zolgensma, nine developed TMA, and one died [85]. In the one patient who died, TMA developed on day 8 after gene therapy. Attempts to improve the patient’s condition were made through hemofiltration and intravenous administration of corticosteroids and eculizumab. There were signs of improvement in TMA after 1 month, but the kidneys did not recover. On day 40, the patient developed sepsis, accompanied by cardiac dysfunction and low blood volume, leading to death. While many factors contribute to a patient’s worsening condition, TMA is the leading cause of SAEs and death.

The mechanism by which TMA is associated with rAAV-based gene therapy is not fully understood, but in addition to close monitoring of patients, current strategies focus on preventive immunosuppression. It was reported that corticosteroids, eculizumab, supportive treatment, and plasmapheresis were effective in treating TMA [86].

### Cardiotoxicity (myocarditis)

Myocarditis and myositis after AAV-based gene therapy are not common adverse events. In a phase Ib trial by Pfizer [87]two DMD patients developed myocarditis following AAV9-based gene therapy; one of the patients died. In the Sarepta therapeutic trial of treatment with the AAVrh74 vector, 1 in 20 patients also developed myocarditis, which subsided after steroid treatment [88]. However, the exact reason for myocarditis caused by AAV vectors is unknown.

## Discussion

In this review, we systematically summarized the results of recent clinical trials of AAV-based gene therapies. These studies not only strongly demonstrated the excellent performance of these therapies in terms of safety and efficacy but also laid a solid foundation for future clinical applications.

### Gene therapy approaches and comparative of AAV-Based treatments

Gene therapy has evolved with various delivery vectors, each offering distinct advantages and limitations. Contemporary viral vector-based gene therapy is achieved by in vivo delivery of the therapeutic gene into the patient by vectors based on retroviruses, adenoviruses (AdV) or AAV.

AdV were the first DNA viruses to undergo full-scale therapeutic development because of their clear biology, genetic stability, efficient gene transfer, and ease of mass production [89]. AdV vectors are mainly used in clinical cancer treatment, and many clinical trials have validated the safety and efficacy of AdV vectors. Adenoviruses have shifted from being primarily used for gene therapy to becoming reliable vectors for delivering vaccines. AdV-derived vectors are now widely recognized as secure and efficient carriers for vaccine components, provoking protective immune reactions against novel elements in both animal and human studies. Their potent immunogenicity makes replicating and non-replicating adenoviruses suitable for cancer vaccine development [90]. Lentivirus (LVs) belong to the orthoretroviridae subfamily of the genus retroviruses. Unlike AdV or AAV vectors, neutralizing antibodies are rarely generated against lentiviral vectors [91]. The most important advantage of LV vectors is their ability to provide long-term and stable gene expression, which is crucial for adolescents or pediatric patients [92]. Nevertheless, LV-based gene therapies face safety challenges including the possible generation of replication-competent LVs during vector production, mobilization of the vector by endogenous retroviruses in the genomes of patients, insertional mutagenesis that may lead to cancer, germline alteration resulting in trans-generational effects, and dissemination of new viruses from the gene therapy patients [92].

AAV-mediated gene transfer has great potential as a therapeutic approach. Most of the currently developed AAV vectors are directed toward monogenic diseases, which belong to the category of rare diseases. AAV vectors are widely favored for in vivo gene therapy due to several advantages, including their ability to transduce both dividing and non-dividing cells, high transduction efficiency in vivo, long-term transgene expression in non-dividing cells, tissue and cell-type specificity, relatively low immunogenicity, non-pathogenic nature, and established clinical safety profile [92]. However, several major challenges limit their widespread application. The maximum recommended insert size for AAV vectors is relatively small, and achieving therapeutic efficacy often requires high systemic doses, which may lead to liver toxicity or immune responses against the capsid, particularly in patients with pre-existing anti-AAV antibodies. Additionally, although AAV vectors rarely integrate into the host genome, the risks of unintended genomic integration or off-target effects still exist [92]. Despite certain disadvantages, AAV vectors nonetheless hold great potential to revolutionize the clinical management of human diseases.

### Recommendations for managing toxicities

The adverse reactions and potential toxicities accompanying AAV-based gene therapies cannot be ignored, including hepatotoxicity, neurotoxicity, TMA, and cardiotoxicity (Table 4). The exact underlying mechanism of these adverse events has not been fully elucidated, but the challenges to therapeutic safety cannot be ignored. Ensuring safety in AAV-based gene therapy demands a comprehensive approach for monitoring its potential impact. AAV-based gene therapies can lead to hepatotoxicity [78, 79]and patients should be screened for liver diseases such as viral hepatitis and alcoholic or nonalcoholic steatohepatitis before treatment. Immunosuppressive therapy (e.g., immunosuppressants), which is currently used in combination with gene replacement therapy, can effectively reduce liver-related toxicity, but it can only partially reduce the severity or incidence of adverse reactions [44, 57]. In any AAV-based gene therapy trial in which a high-dose vector is required, liver function should be carefully monitored to help identify potential SAEs. In several trials in nonhuman primates, intravaginal, intraventricular, macrocisternal, and, to a lesser extent, intravenously transferred high-dose AAV vectors have been shown to cause DGR toxicity [82]. One study revealed that adding microRNA183 targets to the vector reduced transgene expression and toxicity in DRG neurons without affecting transduction in other parts of the primate brain [93]. This strategy may help reduce DRG toxicity and associated morbidity. In clinical trials, we can control the dose of the viral vector, reduce the possible effects of high-dose administration, and carefully design dose increments and safety monitoring programs, in which DRG toxicity can be identified and addressed in a timely manner to increase efficacy and safety. AAV-based gene therapy-induced TMA is a serious and potentially life-threatening complication that may be associated with overactivation of the complement system, which is part of the innate immune system [84]. Cases of TMA have been reported in clinical trials, particularly in gene therapy for SMA. Early identification of TMA is essential for patient management. We should carefully monitor patients’ hematological parameters and organ function after the administration AAV-based gene therapies, follow up after treatment, ensure the appropriate use of corticosteroids and immunosuppressive drugs, and closely monitor TMA markers to adapt the treatment and limit the course of disease.

**Table 4:** Adverse effects/toxicities of AAV-based gene therapy

Adverse effects/toxicities	Pathology/symptom	Proposed mechanism	Treatment
Hepatotoxicity	Elevated liver transaminase Jaundice	Activation of the innate immune and/or complement system	Corticosteroids
DRG toxicity	Axonal degeneration Neuronal damage B cell and T cell infiltration	High doses of AAV vectors	Unknown
TMA	Endothelial injury Platelet aggregation Microthrombus formation	Unknown	Corticosteroids Plasmapheresis Eculizumab
Cardiotoxicity	Unknown	Unknown	Unknown

### Clinical implications and future perspectives

Clinical applications of AAV-based gene therapy have shown considerable promise in treating various genetic disorders. Despite encouraging results, challenges remain in achieving durable therapeutic effects, managing immunogenic responses, and ensuring patient safety. The success of AAV-mediated therapies relies on precise vector design, optimized dosing strategies, and effective immunomodulation protocols to mitigate adverse events such as hepatotoxicity, DRG toxicity, and TMA. From a clinical standpoint, understanding and proactively managing these toxicities is crucial for delivering safer and more efficacious treatments. Close patient monitoring for organ-specific adverse events, combined with timely interventions (e.g., corticosteroid administration), has improved tolerability in several clinical trials. Nonetheless, a broader consensus on standardized monitoring protocols and immunosuppressive regimens is still needed to minimize complications and enhance therapeutic outcomes.

Future advancements in AAV engineering and immunomodulation hold promise for improving both efficacy and safety of gene therapies. Next-generation vectors are being developed with enhanced tissue tropism, reduced immunogenicity, and higher genetic payload capacity. For example, rationally designed capsids that escape preexisting neutralizing antibodies may enable repeated dosing or better transduction in previously restricted patient populations. Parallel efforts in immunomodulatory strategies—including preemptive immune suppression and personalized approaches guided by patient immunoprofiles—could further mitigate toxicity risks. Ultimately, combining these innovations with robust clinical trial designs will be key to translating AAV-based therapies into broadly accessible, safe, and effective treatments for a wide range of genetic diseases.

## Conclusion

AAV-based gene therapies have revolutionized treatment for rare diseases; however, addressing toxicity and improving long-term efficacy remain key challenges.

## Electronic Supplementary Material

Below is the link to the electronic supplementary material.

## Data Availability

All publications that were analysed by the authors to write this review are available in PubMed. The digital object identifiers to locate and download these articles are provided in the reference list.

## Abbreviations

AAV: Adeno-associated virus
FDA: the U.S. Food and Drug Administration
EMA: European Medicines Agency
ITRs: Inverted terminal repeats
ORFs: Open reading frames
AAP: Assembly activating protein
rAAV: recombinant AAV
Sf9: Spodoptera frugiperda cells
rBV: Recombinant baculovirus
rHSV: Recombinant herpes simplex virus
LPLD: Lipoprotein lipase deficiency
IRD: Inherited retinal disease
SMA: Spinal muscular atrophy
AADCD: Aromatic L-amino acid decarboxylase deficiency
HemA: Hemophilia A
HemB: Hemophilia B
DMD: Duchenne muscular dystrophy
ILM: Internal limiting membrane
RPE: Retinal pigmented epithelium
IV: Intravenous
CNS: Central nervous system
IPa: Intraparenchymal
PD: Parkinson’s disease
ICV: Intracerebroventricular
ICM: Intracisterna magna
CSF: Cerebrospinal fluid
LCA: Leber’s congenital amaurosis
RP: Retinitis pigmentosa
BCVA: Best corrected visual acuity
DDC: Dopamine decarboxylase
SMN1: Spinal cord motor neuron 1
SAEs: Serious adverse events
CD: Canavan disease
TSD: Tay‒Sachs disease
GD: Gaucher disease
FD: Fabry disease
ALT: Alanine aminotransferase
DRG: Dorsal root ganglion
ALS: Amyotrophic lateral sclerosis
SOD1: Superoxide dismutase 1
TMA: Thrombotic microangiopathy
AdV: adenoviruses
LVs: Lentivirus
